# Assessment of Lingual Tactile Sensitivity in Children and Adults: Methodological Suitability and Challenges

**DOI:** 10.3390/foods9111594

**Published:** 2020-11-03

**Authors:** Marta Appiani, Noemi Sofia Rabitti, Lisa Methven, Camilla Cattaneo, Monica Laureati

**Affiliations:** 1 Department of Food, Environmental and Nutritional Sciences (DeFENS), University of Milan, 20133 Milan, Italy; marta.appiani@unimi.it (M.A.); noemi.rabitti@unimi.it (N.S.R.); monica.laureati@unimi.it (M.L.); 2 Department of Food and Nutritional Sciences, University of Reading, Whiteknights, Reading RG6 6AP, UK; l.methven@reading.ac.uk

**Keywords:** Von Frey filaments, gratings orientation test, child, tactile sensitivity, age-related differences, food texture, texture preference, food neophobia

## Abstract

Few methodological approaches have been developed to measure lingual tactile sensitivity, and little information exists about the comparison between children and adults. The aims of the study were to: verify the cognitive and perceptive suitability of Von Frey filaments and a gratings orientation test in children of different ages; compare lingual tactile sensitivity between children and adults; investigate the relationships between lingual tactile sensitivity, preference and consumption of foods with different textures and level of food neophobia. One hundred and forty-seven children aged 6–13 years and their parents participated in the study, in addition to a separate sample of seventy adults. Participants filled in questionnaires, and lingual tactile sensitivity was evaluated through filaments and gratings. Results showed that gratings evaluation was more difficult than filaments assessment but enabled a better separation of participants according to their performance than filaments. R-indices from filaments were not correlated with those of gratings, suggesting that the tools measure different dimensions of lingual tactile sensitivity. No differences were found in lingual tactile sensitivity between children and adults, nor between children of different ages. Food neophobia was negatively associated with preferences of hard foods in children. Although a multifactor analysis concluded that neither texture preferences nor food consumption were strongly correlated with lingual tactile sensitivity, there was a weak but significant positive correlation between lingual tactile sensitivity to the finest Von Frey filament and food neophobia in the youngest age group, indicating that children with higher levels of food neophobia are more sensitive to oral tactile stimuli. Suitable child-friendly adaptations for the assessment of lingual sensitivity in children are discussed.

## 1. Introduction

Food texture is multifactorial, the perception of which encompasses many sensory dimensions, ranging from touch to visual and auditory sensations [1].

Over the past decades, considerable advances have been made in the understanding of cutaneous tactile perception [2,3,4,5]. In non-hairy skin, four specialized mechanoreceptor nerve endings have been identified to convey specific sensations of touch. The Merkel cell discs are in the basal layer of the epidermis and respond to light pressure. Meissner corpuscles are localized in the dermal papillae and respond to movement, as well as light touch. Ruffini endings and Pacinian corpuscles are both located in deeper layers of the dermis. Ruffini endings have been associated with sensations of stretch, while those terminating in Pacinian corpuscles detect high-frequency vibration [6]. Regarding the peripheral neural mechanisms underlying tactile sensation in the human tongue, only three classes of low-threshold mechanoreceptors have been identified, as there is no evidence of Pacinian corpuscles underlying the dorsal lingual mucosa [7].

Compared to other senses such as taste and smell, oral tactile perception has received little attention for many years, despite being an important driver of food acceptance and rejection, especially in children [8,9,10].

Recently, this sensory modality is being systematically and extensively investigated in view of its importance in food product development and with consideration of individual variations in texture perception and preference, including age-related differences. For instance, it has been shown that individuals vary in the way they manipulate food in their oral cavity, and these different mouth behaviors are predictive of food preferences and choices [11]. Moreover, children greatly differ from adults in texture preference [12,13], probably due to developments of the mouth muscles, jaw and teeth, as well as innervation of taste buds [8,13,14,15]. Children tend to prefer soft and homogeneous foods rather than lumpy or gummy foods with pieces inside [8,10,16]. Therefore, it is important to optimize food texture in order to promote children’s acceptance of products containing particles [17,18,19], such as plant-based or fiber-enriched food [16].

The importance of texture in children’s rejection of certain foods is also underlined by the fact that a higher tactile sensitivity is associated with food neophobia and pickiness, which are both high during childhood [18]. Food neophobia is defined as the rejection of novel or unknown foods, while picky/fussy eating is the rejection of both familiar and unfamiliar foods [20]. Both constructs are known to be important barriers to healthy eating. In this respect, children who tend to reject foods with particles or lumpy food are more often picky eaters or food neophobic [21]. This knowledge emphasizes the importance of studying lingual tactile sensitivity in the context of fussy or picky eating behavior, since it has been suggested that a gradual exposure to changes in texture from an early age could possibly help children to overcome this maladaptive trait [10,18], increasing fish, fruit and vegetable consumption and making novel foods more acceptable [22,23,24,25].

Although texture has been shown to play an important role in food choice and health status, so far, few methodological approaches have been developed to measure tactile sensitivity at the oral level, and little information exists about the comparison between children and adults.

One method for the assessment of lingual tactile sensitivity was proposed by Essick [26] and consists of the recognition of letters embossed on Teflon strips positioned on the tongue of blindfolded subjects. Although this method has been successfully used with children aged 7–10 years to explore age-related differences with their mothers [13], it may suffer from some psychological errors that can alter the repeatability and reliability of the measurements [27]. First of all, not all letters are recognizable in the same way; for instance, it is easier to recognize letters such as “I” than other more complex letters, such as “Z” and “W”. This method is also affected by the cognitive development of the individuals involved and, for this reason, might not be suitable for young children who are not yet familiar with the alphabet. Moreover, this method is very limiting when cross-cultural studies involving countries that do not use the Latin alphabet are conducted [28].

The Von Frey filaments are a tool commonly used in the medical field to assess the tactile sensitivity of hands and feet in order to diagnose diseases such as dysesthesia (i.e., abnormally increased sensitivity to touch stimuli) and hypesthesia (i.e., abnormally decreased sensitivity to touch stimuli) [29]. It consists of nylon filaments of different thickness, which allow the discrimination of small touches or slight pressures on the skin. This approach was used recently to measure oral tactile sensitivity (i.e., the tongue) but only in adults [28,30,31,32].

The gratings orientation test is another method used to assess tactile sensitivity on the tongue. This method consists of square blocks engraved with ridges (gratings) on their surface. The blocks are positioned on the tongue of blindfolded subjects, who are asked to recognize the orientation (horizontal vs. vertical) of the ridges. This approach was used by Van Boven et al. [33] to assess lingual tactile sensitivity in a group of adults; however, it is not known whether it is suitable for children.

Due to the paucity of methodological approaches to measure tactile sensitivity at the oral level in the pediatric population, and in view of the relation that might be envisaged between tactile sensitivity, food texture preference and consumption, the main aim of the present study was to verify the suitability of the Von Frey filaments and the gratings orientation test in children of different ages (6–13 years). Children’s lingual tactile sensitivity was compared to that of adults that were involved as a control group. A secondary goal was to investigate the relationships between lingual tactile sensitivity, preference and consumption of foods with different textures and food neophobia.

## 2. Materials and Methods

### 2.1. Participants

One hundred and forty-seven children aged 6–13 years participated in the study, alongside their parents, who completed questionnaires about their children’s eating habits and consumption frequency of different food categories. A separate sample of seventy adults aged 19–33 years was also involved. Henceforth, we will refer to the two separate groups of adults as parents and adults. The characteristics of the study participants are reported in Table 1. Of the 147 children who participated in the study, only 65 parents (44.2%) filled out the questionnaires. Mothers (76.9%) completed the questionnaire more frequently than fathers.

Children were recruited from two schools in the metropolitan area of Milan (Italy). Parents were informed about the procedures of the study and were asked to sign an informed consent when they agreed on their child’s participation. Invited children were informed about the test and gave verbal consent. Children without a signed informed consent or declining participation verbally were excluded from the study.

Adults were recruited among students and staff of the Faculty of Agriculture and Food Sciences of the University of Milan. Informed, written consent was obtained from all adults on the test day.

This study was approved by the Ethics Committee of the University of Milan (n. 48/19) and conducted in accordance with the Declaration of Helsinki.

### 2.2. Experimental Procedure

Children and adults attended one study session, completing two different tasks: (1) completion of questionnaires and (2) lingual tactile sensitivity assessment using Von Frey filaments and the gratings orientation test. The whole task lasted about 20–30 min for children and 30 min for adults. In the case of some 6-year-old children, more time was required, and sometimes, although rarely, it was necessary to interrupt the experimental session and to continue it after a break.

For the group of children and adults, the assessment of the lingual tactile sensitivity was interspersed between the completion of questionnaires to prevent the effects of fatigue, especially in children. The tasks performed by the study participants are reported in Table 1.

Children were tested in a familiar and ecological environment (i.e., their school), while the adults were tested at the Sensory & Consumer Science Laboratory of the University of Milan.

#### 2.2.1. Questionnaires Completed by Children

Children completed the questionnaires using tablets. This choice was made in an attempt to create a game-like situation, thus keeping pupils’ attention high [36]. Since consumer testing with children requires a specifically designed introduction to the methodologies and more extensive training [37], the research team visited the classes and carefully explained the procedures to the children prior to the beginning of the test. The day of the session, groups of 3 to 4 children were accompanied by 3 to 4 female experimenters in a quiet room inside the school where they were individually seated so that they were not facing each other and could not interact. Each child was interviewed by one experimenter. The children filled out the questionnaires themselves. Some younger children (6 years old) completed the questionnaires with the aid of the experimenter, who read them the instructions and questionnaires and sometimes helped them to enter their answer.

Children answered questions related to their date of birth, age and sex. In order to assess children’s texture preferences, they completed the Child Food Texture Preference Questionnaire (CFTPQ) [12]. This questionnaire consists of 17 pictures of food pairs. Within each pair, foods differ in their texture (e.g., chocolate bar vs. chocolate mousse) but, also, for the presence/absence of pieces or particles (e.g., yoghurt with fruit pieces vs. yoghurt without fruit pieces). For each pair, children were asked to indicate the preferred item (“Which product do you prefer?” forced choice answer), and later, they were asked about their familiarity with each item (“Have you ever tasted this product?” Yes/No).

Children also completed a validated, child-friendly version of the Food Neophobia Scale [34] to investigate their neophobic traits. This questionnaire consists of eight items, 4 related to neophilic and 4 related to neophobic attitudes. Children scored them using a 5-point scale with facial expressions representing different degrees of agreement (“Very false for me” = a frown face with both thumbs down, “False for me” = a frown face with one thumb down, “So-so” = a neutral face with no thumbs shown, “True for me” = a smiley face with one thumb up and “Very true for me” = a smiley face with both thumbs up). The answers to the items of the Food Neophobia Scale (FNS) were summed up (after reversing the scores of the neophilic items) to have a food neophobia score ranging from 8 to 40. A higher score indicates a higher level of food neophobia.

#### 2.2.2. Questionnaires Completed by Parents

The parents of the participating children were invited to complete an online questionnaire to obtain sociodemographic information, eating habits of their child and complementary information about their child and themselves. Parents reported also on their own age and sex.

Moreover, parents completed a food consumption frequency questionnaire (FFQ) that focused on their child’s intake of 41 foods, inspired by a questionnaire previously used by Laureati et al. [12]. Different categories of food were chosen: (1) bakery products, (2) fruits and vegetables, (3) potatoes, (4) dairy products and (5) sweet products. Within each category, foods with different textures were chosen (Table 2). Each food was represented by a picture to make it more specific for the parents. For each item, parents had to indicate how often their child consumed it in the last year. Answering options were: “never”, “1 to 2 times a year”, “3–11 times a year”, “once a month”, “2 to 3 times a month”, “once a week”, “2 times a week”, “3 to 4 times a week”, “5 to 6 times a week” and “every day”. The frequency of consumption for each food item (FFQ) was converted into the number of times each food was consumed during the year. The scores were calculated as follows: 0 = never, 1.5 = 1 to 2 times a year, 7 = 3–11 times a year, 12 = once a month, 30 = 2 to 3 times a month, 52 = once a week, 104 = 2 times a week, 182 = 3 to 4 times a week, 286 = 5 to 6 times a week and 365 = every day.

#### 2.2.3. Questionnaires Completed by Adults

Adults completed the CFTPQ as described for the children but without answering the question on familiarity, as it was supposed that all the food items would be well-known by the adults [6]. Food neophobia was measured using the Food Neophobia Scale (FNS) [35], validated in Italian as described by Laureati et al. [38]. The FNS consists of ten statements, of which five are positively, and the other five negatively, worded, each measured on a 7-point scale ranging from 1 = “strongly disagree” to 7 = “strongly agree”. As for the children, the answers to the items of the FNS were summed up (after reversing the scores of the neophilic items) to have a food neophobia score ranging from 10 to 70. Interpolation was performed to calculate comparative scores with those of the children. Finally, the adults filled out the same FFQ as described for the parents.

#### 2.2.4. Lingual Tactile Assessment

Tactile sensitivity tests were run with children and adults. Two different tools were used: Von Frey filaments and gratings. The experimenters were trained in the use of both instruments at a preliminary stage of the actual experiment. As far as the filaments were concerned, the experimenters were trained about the pressure to be exerted by evaluating a group of volunteers. Since the filaments were very thin, after several tests, it was decided to conduct the test sitting side-by-side with the subject and not frontally and with a dark background; in this way, it was easier for the experimenters to see when the filament touched the surface of the tongue. With regards to the gratings orientation test, the experimenter calibrated the pressure to be exerted (100 g) on the tongue using a scale and a sponge.

The approach applied to assess the lingual tactile assessment is based on signal detection theory (SDT), originally designed to describe the ability of an observer to recognize whether the source of a voltage change is noise or signal plus noise [39]. Soon afterward, SDT was adopted by cognitive scientists to measure human decision-making in perceptual studies, e.g., [32,40,41] and sensory sensitivity [42,43]. SDT’s greatest advantage over classical psychophysical methodologies is the separate determination of parameters reflecting sensory and non-sensory factors and that it can be applied to any kind of stimulus (e.g., images, odors, sounds, food samples and water solutions), as well as in different fields (e.g., psychology, diagnostics of any kind and quality control) [44]. Moreover, SDT enables the calculation of indexes (e.g., R-index and d’) that, unlike the traditional discriminant sensory methods, provide significance checks only, given the size of the discrimination level between stimuli. Additionally, these indexes are a signal detection measure and are thus free of what is called a response bias or criterion variation [45]. Moreover, traditional discriminant methods are critically influenced by subject cooperation and may therefore be influenced by boredom, mental fatigue or distraction [46], especially when the pediatric population is involved.

##### Von Frey Filaments

This test used fine Von Frey filaments (Aesthesio^®^: Precise Tactile Sensory Evaluator, DanMic Global, LLC, San Jose, CA, USA). This tool consists of a set of 20 filaments of different sizes that apply a force ranging from 0.008 g to 6.0 g. For the present experiment, we chose the two smaller filaments that apply a 0.008-g and a 0.02-g force, respectively. The choice of the thickness of the two filaments was made in accordance with Cattaneo et al. [28], who used these tools to evaluate lingual tactile sensitivity in adults. Prior to the evaluation, subjects were informed about the purpose of the test showing the two filaments. The larger filament (0.02 g) was first used on the children’s hands, both to reassure them that they were not dangerous and/or painful and to help train them in the method.

For each filament, this test was conducted 24 times, 12 times with true touch (in a balanced order on left and right sides of tongue) and 12 times without any touch. The presentation order of the true touch and false touch was balanced within each filament size. Children were presented first with the series of the 24 thinner filaments (0.008 g) and then with the series of the 24 thicker ones (0.02 g). For each filament size, the participants wore a blindfold for the first set of 12, then removed it and had a sip of water before replacing it and doing the next set of 12. The subjects were aware that some touches were true and some were false. Immediately after each touch, the subject was asked the following questions: “Did you feel a touch or not?” (Yes/No) and “Are you sure or unsure?” (Sure/Unsure). After each subject evaluation, filaments were disinfected using cotton discs soaked in a 2% disinfectant solution (Amuchina, Acraf S.p.A, Ancona, Italy).

##### Gratings Orientation Test

This test used machine-cut squares (1 cm^2^) from polytetrafluoroethylene (PTFE) rods. Each square had a 0.5-mm height and was held by a narrow cylindrical rod (2 cm long). There were six different types of squares varying in groove/bar widths (0.2, 0.25, 0.5, 0.75, 1.00 and 1.25 mm), as shown in Table 3. For each square, the groove and bar widths were identical; groove depths increased as groove widths increased (Table 3). These tools were specifically made for this study by the University of Reading (University of Reading, Whiteknights Campus, Reading, UK) and the University of Copenhagen (Department of Food Science, Section for Food Design and Consumer Behaviour, Copenhagen, Denmark). An alternative would have been to use commercially available JVP domes (Stoelting Co, Wood Dale, IL, USA), which are acrylic probes with equidistant bar and groove widths nominally equal to 0.35, 0.5, 0.75, 1.00, 1.25, 1.5, 2.00 and 3.00 mm and designed to measure the cutaneous tactile sensitivity on fingers. However, considering that the average spatial resolution at the tongue is around 0.58 mm [33] and that large individual variability in lingual tactile perception exists, we decided to produce custom-made gratings with additional groove widths below 0.5 mm (i.e., 0.20 and 0.25 mm) in order to have tools with sizes more suitable to assess oral tactile sensitivity.

**Figure 1:**
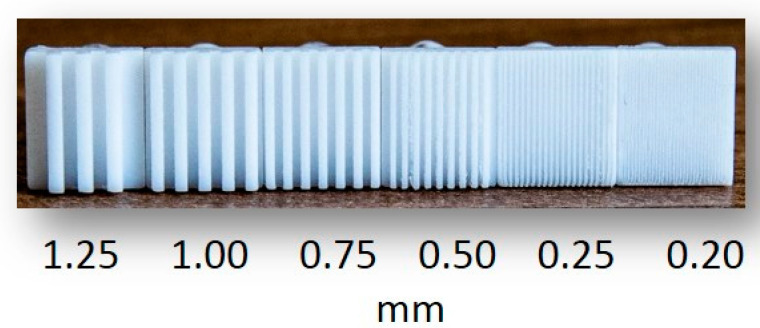
Gratings with decreasing sized grooves/bars (0.20 mm on the right). With children, only three gratings were used (0.75 mm, 1.00 mm and 1.25 mm), while, for adults, the complete set was used.

All six different-sized groove widths were tested with the adults. In order to reduce fatigue in children during the task, and following an initial pilot study with children, it was concluded that 0.75 mm was appropriate (below this grating size, children’s performance was very low) as the smallest grating size for this target population, and hence, only three different-sized groove widths (0.75, 1.00 and 1.25 mm) were tested with them. The 6 different squares are depicted in Figure 1.

Prior to the evaluation, the experimenter explained to the subject what the test consisted of, showing the tools. A simulation of the test with the 1.25-mm grating was made without the subject wearing the blindfold to make it clear how to perceive the horizontal/vertical orientation. Then, the volunteer was blindfolded, and each grating was presented 6 times (three in horizontal and three in vertical). The presentation order of the different grating sizes was balanced, as well as the orientation (horizontal vs. vertical). After each touch, the volunteer was asked: “What do you think is the orientation of the instrument, horizontal or vertical?” (Horizontal/Vertical). Since, for children, especially the youngest ones, the concepts of “horizontal” and “vertical” were not evident, the experimenter, prior to the beginning of the test, showed the two possible orientations of the grooves and, then, after each touch, asked “Were the grooves horizontal or vertical?” whilst encouraging the child to use his/her hand to indicate the orientation. The subject was also asked to report his/her degree of sureness (“Sure” or “Unsure”). After each subject evaluation, gratings were immersed in a 2% disinfectant solution (Amuchina, Acraf S.p.A, Ancona, Italy) for 15 min; after which, they were thoroughly rinsed with tap water and brushed with a toothbrush. As each grating was presented only 6 times, a test-retest assessment was performed approximately 1 month later on the children (*n* = 121). Spearman’s correlation showed positive and significant associations between the first and second assessments for all gratings tested (G0.75: ρ = 0.857, *p* < 0.001, G1.00: ρ = 0.823, *p* < 0.001 and G1.25: ρ = 0.906, *p* < 0.001). For practical constraints, the test-retest assessment was not possible with adults.

### 2.3. Data Analysis

The SAS/STAT statistical software package version 9.4 (SAS Institute Inc., Cary, NC, USA) and XLSTAT (version 2019.2.2, Addinsoft, Boston, MA, USA) were used for the data analysis. Effects showing a *p*-value of 0.05 or lower were considered significant.

#### 2.3.1. Lingual Tactile Sensitivity

Individual tactile sensitivity for each instrument (i.e., filaments and gratings) and for each size (2 sizes for filaments and 3–6 sizes for gratings) were expressed by R-index [44]. R-Index is a nonparametric statistic that can be computed from a variety of methods, including sensitivity measurements in psychophysics, sensory difference testing, hedonic scaling and preference tests. R-index is an estimated probability of correctly identifying a target stimulus (the signal) when presented pairwise with a second stimulus (the noise). The index is based on SDT; it is free of the response bias that can invalidate difference testing protocols, and it represents a measure of the subjects’ ability to discriminate (i.e., sensitivity) between a stimulus and a blank (for review, see [45]). This index has been successfully used in previous studies to measure lingual tactile sensitivity [28,32].

Since, for each tool and size, each subject was presented with both the signal (S) and the noise (N) and indicated whether he/she was “sure” or “unsure” of his/her decision, four response options could occur for both signal and noise: “S-sure”, “S-unsure”, “N-unsure” and “N-sure”. For the gratings orientation test, the signal and the noise correspond to the horizontal-vertical orientation of the square, while, for the filaments, the signal and noise are the true and false touch, respectively. For each individual, and for all filaments and gratings, the frequencies of answer were collected in a response matrix (Table 4) and used to calculate the R-index [44], where a–h are integers taking values between 0 and 12 in the filaments approach and values between 0 and 3 in the gratings approach. The total number of signals presented will be (a + b + c + d). Similarly, the total number of noise presentations will be (e + f + g + h).

(1) R−index=a(f+g+h)+b(g+h)+ch+12(ae+bf+cg+dh)(a+b+c+d)(e+f+g+h)

R-index values range from 0 to 1. A higher R-index value indicates a better discrimination. In the present study, the cut-off values for discrimination were set to, respectively, 0.6323 and 0.7426 for filaments and gratings, according to the one-sided R-index critical values for α = 0.05 [47].

R-index frequency distributions by age group for all tools (filaments and gratings of all sizes) were calculated and checked for normality. Q-q plots were inspected, and residual distributions were also checked for normality. In all cases, raw data and residuals distributions were not normal, according to the Shapiro-Wilk test (W range: 0.86–0.96, *p* < 0.05).

In order to investigate age-related differences in lingual tactile sensitivity, children were grouped in four age groups: 6 to 7 years old (*N* = 38), 8 to 9 years old (*N* = 43), 10 to 11 years old (N = 32) and 12 to 13 years old (*N* = 34). Age groups were compared with adults (*N* = 70). Age-related and sex-related differences in tactile sensitivity, expressed as R-index values, were statistically evaluated with Kruskal Wallis and Mann-Whitney tests, respectively, with Dunn-Bonferroni correction for multiple comparisons.

The relationship between lingual tactile sensitivity measured by Von Frey filaments and the gratings orientation test was examined using Spearman’s correlation coefficients and principal component analysis (PCA) performed on individual R-index values (tactile sensitivity measures fitted as variables and subjects as observations). To explore the relationship between lingual tactile sensitivity, food neophobia, texture preference and food frequency of consumption, a multifactor analysis (MFA) was used. For this analysis, the data from the 0.02-g force filament (F0.02) was excluded, as it was more highly skewed than data from the remaining four tools, and significant data outliers were removed (identified by Grubbs outlier test as five F0.008 data points and no grating data points from the three gratings that were used to test both adults and children: G0.75, G1.0 and G1.25). For MFA, data were incorporated as three tables: lingual tactile assessment (4 variables: F0.008, G0.75, G1.0 and G1.25); preference measured (2 variables: CFTPQ and FNS scores) and food consumption frequency, FFQ (13 variables corresponding to the texture versions in Table 2). Active tables were set as the lingual tactile assessment and preference measures, with the FFQ as a supplementary table.

#### 2.3.2. Food Texture Preferences

Individual CFTPQ indices were calculated to express food texture preference. According to Laureati et al. [12], when the hard/particulate version of a food pair was preferred, a score of 2 was given. When children preferred the soft/smooth version of a food pair, they received a score of 1 for that pair. Only food pairs where both of the items were familiar to the child (i.e., were tasted before) were used for the CFTQP index calculation for that child. Children with <8 valid pairs (which was approximately 50% of the total pairs) were removed from the calculations. Thus, each participant could theoretically obtain a score ranging from 8 to 34. To make the score more discriminative and easier to interpret, the CFTPQ index was calculated by the following formula:

(2) $$\mathrm{CFTPQ}\mathrm{index}=\left[\left(\frac{\mathrm{Sum}\mathrm{of}\mathrm{the}\mathrm{scores}\mathrm{of}\mathrm{the}\mathrm{valid}\mathrm{pairs}}{\mathrm{Total}\mathrm{number}\mathrm{of}\mathrm{valid}\mathrm{pairs}}\right)-1\right]*100$$

This resulted in a CFTPQ index that ranged from 0 to 100, with higher scores representing a preference for the harder foods category. A similar calculation was done for the group of adults considering all of the 17 food pairs as valid.

## 3. Results

### 3.1. R-Indices Distributions

**Figure 2:**
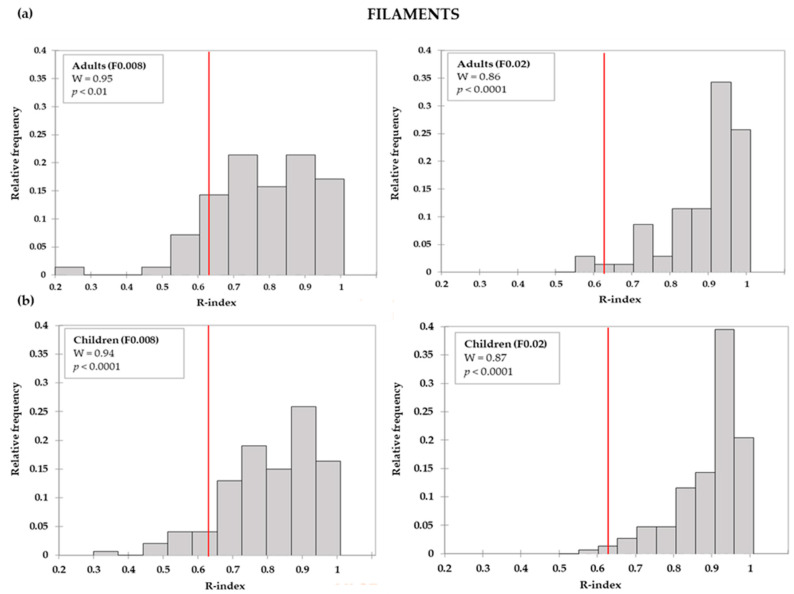
Adults (**a**) and children (**b**) R-index distributions for filaments (F). The vertical lines indicate the R-index cut-offs for discrimination (*R* = 0.63). The boxes show the Shapiro-Wilk test (W) statistics and respective level of significance.

**Figure 3:**
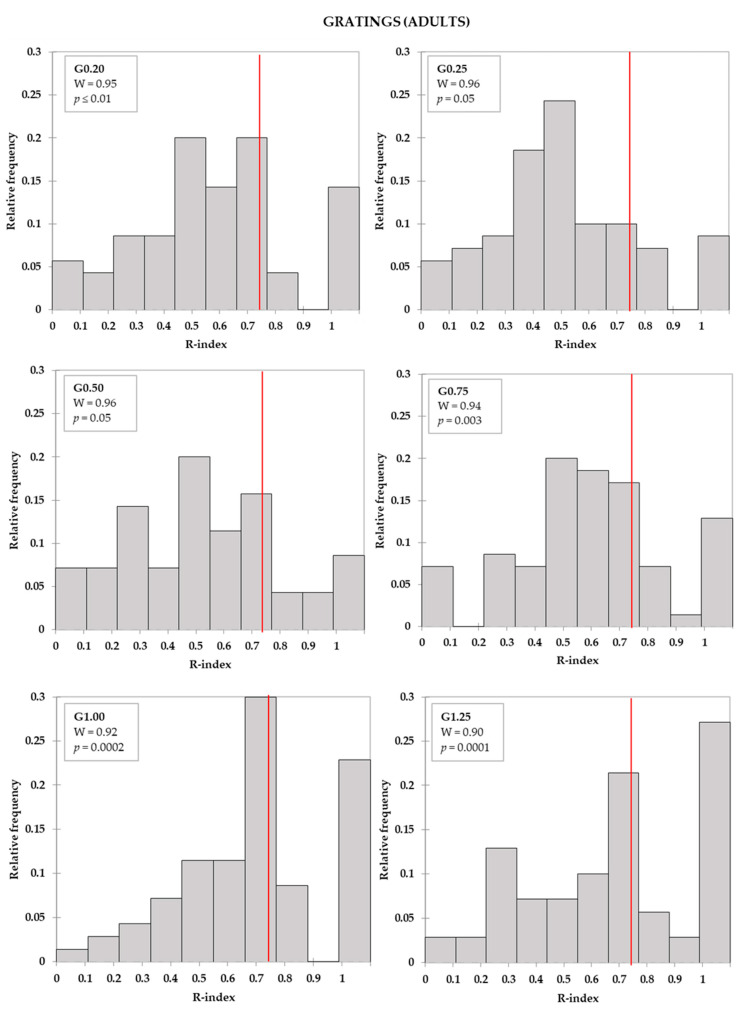
Adult R-index distributions for gratings (G). The vertical lines indicate the R-index cut-offs for discrimination (*R* = 0.74). The boxes show the Shapiro-Wilk test (W) statistics and respective level of significance.

**Figure 4:**
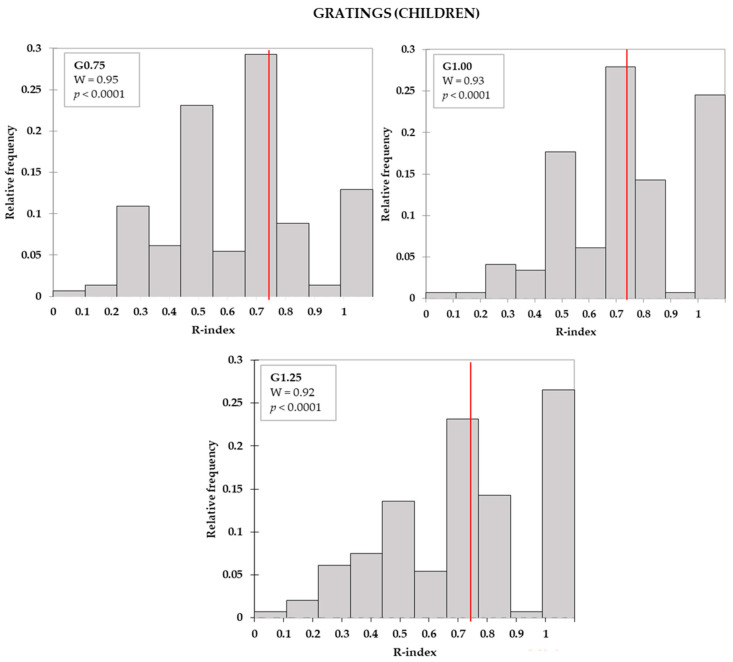
Children’s R-index distribution for gratings (G). The vertical lines indicate the R-index cut-offs for discrimination (*R* = 0.74). The boxes show the Shapiro-Wilk test (W) statistics and respective levels of significance.

R-indices distributions by tool and by age group are depicted in Figure 2, Figure 3 and Figure 4. For both adults (Figure 2a) and children (Figure 2b), R-indices from the filaments were negatively skewed, indicating that most of the subjects were able to correctly identify the touch on the tongue (high R-indices). Concerning gratings, these tools enabled a better separation of subjects according to the ability to correctly identify the groove orientations. Moreover, it could be seen that, with the increase of the groove size, the proportion of subjects with higher R-index increased, although modestly, for both adults (Figure 3) and children (Figure 4).

### 3.2. Age- and Sex-Related Differences in Lingual Tactile Sensitivity

**Figure 5:**
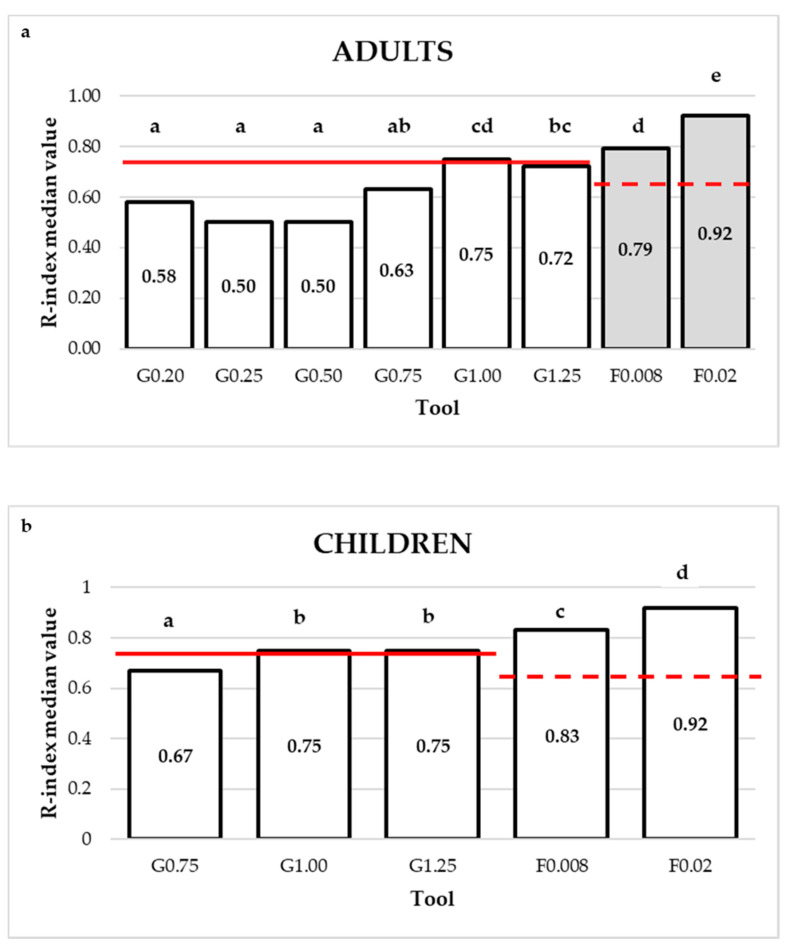
(**a**,**b**). R-index median values by age and tool. The full and dashed lines indicate, respectively, the R-index discrimination cut-offs for gratings (G) (*R* = 0.74) and filaments (F) (*R* = 0.63). Values marked with different letters are significantly different (Dunn-Bonferroni test, *p* < 0.05).

R-index values by age group and by tool are reported in Figure 5a,b. For both adults (Figure 5a) and children (Figure 5b), the discrimination ability increased according to the size of each of the two tools. Moreover, both age groups (adults: *K* = 14.07, *p* < 0.0001 and children: *K* = 9.49, *p* < 0.0001) performed significantly better with filaments than gratings. In fact, for both age groups, R-indices for both filaments were above the discrimination cut-off of 0.63, while, for gratings, both adults and children were, on average, able to correctly discriminate the orientation of the grating’s grooves from grating 1.00 (R-index values higher than the cut-off of 0.74 for those tools).

**Figure 6:**
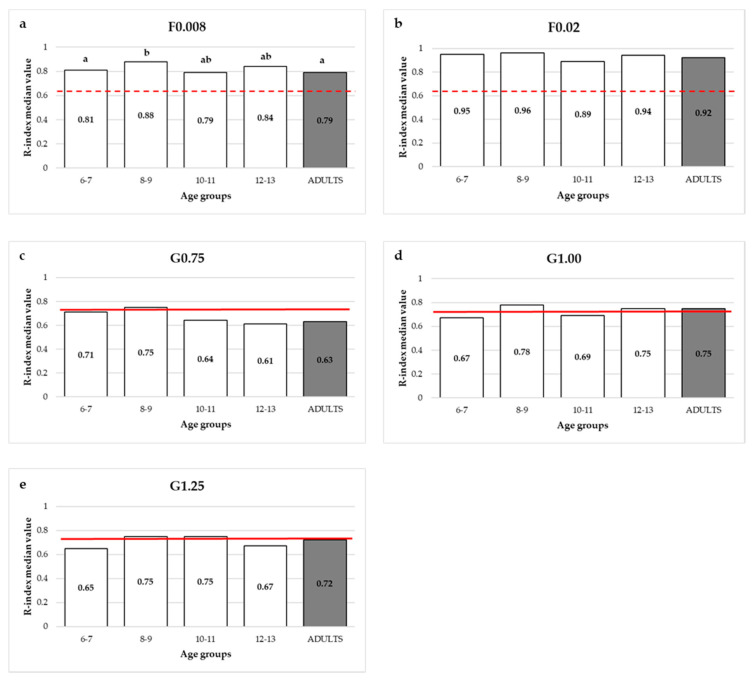
(**a**–**e**). R-index median values by age and tool. The full and dashed lines indicate, respectively, the R-index discrimination cut-offs for gratings (G) (*R* = 0.74) and filaments (F) (*R* = 0.63). Different letters indicate significant differences (Dunn-Bonferroni, *p* < 0.05).

In order to investigate age-related differences in lingual tactile sensitivity (R-index), the four age groups of the children (6 to 7, 8 to 9, 10 to 11 and 12 to 13 years old) were compared with the adults (Figure 6a–e). As regards gratings, the performances in the three gratings (G0.75, G1.00 and G1.25) used with both adults and children were compared.

The Kruskal-Wallis results showed that the performances of the children and adults were significantly different only for the finest filament, F0.008 g (*K* = 11.3; *p* = 0.02). Children aged 8 to 9 years (median R-index = 0.88) were significantly more sensitive than children aged 6 to 7 years (median R-index = 0.81; *p* = 0.004) and adults (median R-index = 0.79; *p* = 0.002) (Figure 6a).

Considering sex, according to the Mann-Whitney test, significant differences in lingual tactile sensitivity were found only for the greatest grating size, G1.25, where adult women performed significantly less well than both adult men (*p* = 0.016) and girls (*p* = 0.025) (data not shown).

### 3.3. Relationship between the Tools

The analysis was conducted on the tools that were tested on both adults and children (both filaments and for groove sizes ≥0.75 mm) and considering the total sample of the participants (*n* = 217), since the analyses performed separately for adults and children provided very similar plots.

**Figure 7:**
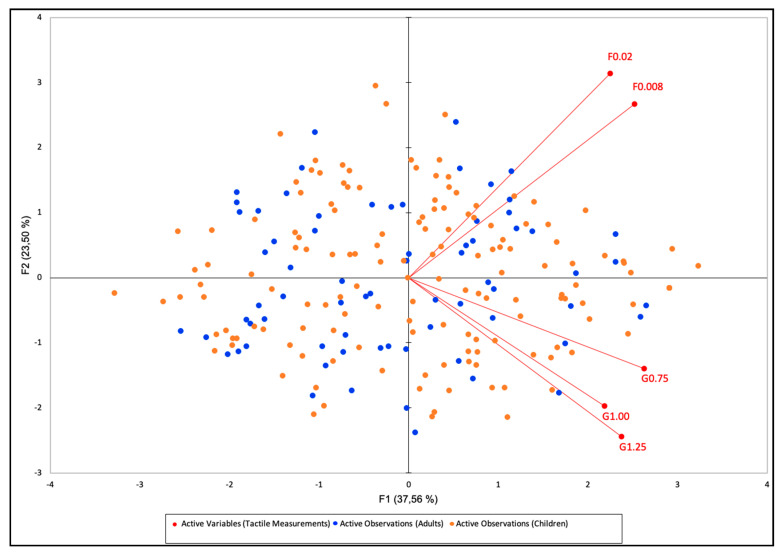
Biplot from the principle component analysis (PCA) performed on individual R-index values over all subjects (*n* = 217).

The biplot depicting the positioning of the R-index values by tool is reported in Figure 7. The total explained variance was 61.06%, with PC1 contributing for 37.56% and PC2 for a further 23.50%. All tools are positioned in the positive part of PC1, while PC2 separates the two filaments (positioned near to each other on the positive part of PC2) from the three gratings (positioned near to each other in the negative part of PC2).

The correlations between the variables that the PCA was based upon were further investigated through Spearman’s coefficients (Table 5).

The correlations between R-values for filaments of different sizes, as well as between gratings of different sizes, were positive and highly significant (*p* < 0.0001). The correlation between filaments and gratings was less evident and seemed to increase with the decreasing of the groove width and filament thickness.

### 3.4. Relationship between Lingual Tactile Sensitivity, Texture Preference, Food Neophobia and Food Consumption Frequency

**Figure 8:**
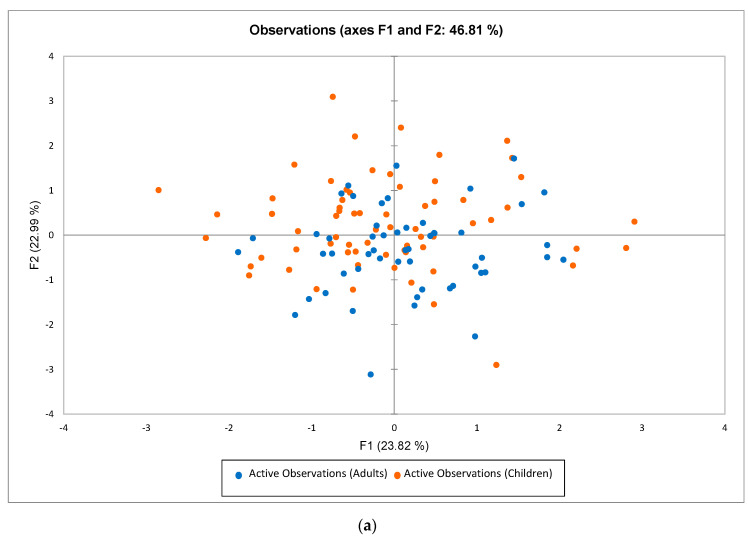

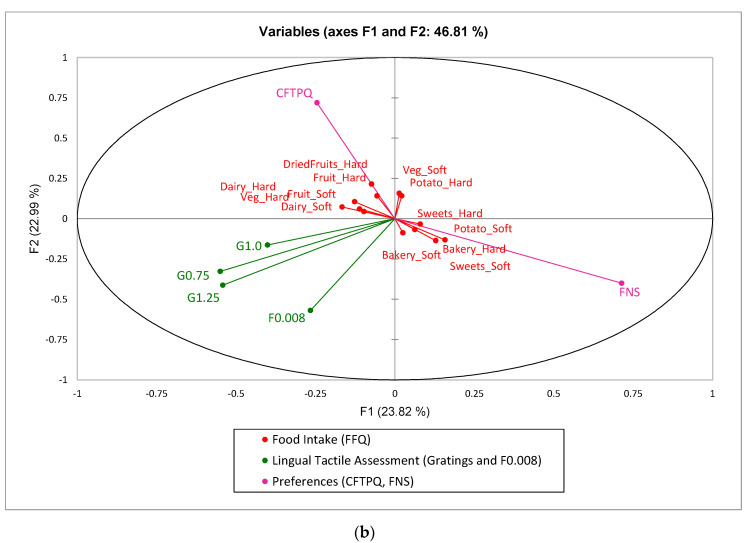
(**a**,**b**). Observations and variable plots from a multifactor analysis (MFA) performed on lingual tactile sensitivity (4 variables: F0.008, G0.75, G1.0 and G1.25); preference measured (2 variables: Child Food Texture Preference Questionnaire (CFTPQ) and Food Neophobia Scale (FNS) scores) and the food frequency of consumption, FFQ (13 variables). Active tables were set as the lingual tactile assessment and preference measures, with the FFQ as a supplementary table.

The MFA depicting the relationship between lingual tactile sensitivity, food neophobia, texture preference and food frequency of consumption is shown in Figure 8a,b. The observations plot (Figure 8a) showed that children (orange dots) and adults (blue dots) were both distributed across the plot, with no obvious separation of adults and children.

Considering the variables plot (Figure 8b), the total explained variance was 46.81%. Along F1 (23.82% explained variance), R-indices from all tools (filament F0.008 and gratings of different sizes) are positioned near in the lower left quadrant and are opposed to the FNS (i.e., high food neophobia). Along F2 (22.99% explained variable), the CFTPQ index, which reflects a preference for hard food, is opposed to the FNS. This positioning indicates that subjects with high FNS (high neophobia) have lower CFPTQ scores (i.e., they prefer soft foods). CFTPQ is, in turn, positioned near to most of the hard versions of foods (i.e., fruits, dairy, dried fruits and nuts and vegetables) and, also, to two soft food versions (i.e., fruits and dairy), indicating that subjects with preferences for hard foods consume hard foods more frequently. However, in general, the consumption of hard and soft versions of food was poorly separated on F1, as well as on F2. The FNS was positioned near to the consumption of starchy (bakery products and potatoes) and sweet foods.

The correlations between the variables that the MFA was based upon were further investigated using Spearman’s correlation. Indeed, there was no overall significant correlation between lingual tactile sensitivity and overall texture preference. Similarly, there was no significant correlation between lingual tactile sensitivity and food neophobia overall, despite the opposed location on the MFA plot, implying that a high lingual tactile sensitivity was not related to a lower FNS, and that would have been an unexpected finding. Indeed, when investigating within age groups, a weak but significant and positive correlation was found between lingual tactile sensitivity (F0.008) and food neophobia in the youngest age group (6 to 7 years: ρ = 0.32; *p* = 0.05), indicating that children with higher levels of food neophobia are more sensitive to lingual tactile stimuli. There were no other significant correlations between lingual tactile sensitivity and food neophobia by age group and by tool.

## 4. Discussion

Texture plays an important role in food quality and acceptability, which mutually affect individuals’ food preferences and choices [48], especially among pediatric populations [10]. While it has been established that individual differences in taste and smell function have a strong influence on food preference and choice in both adults and children, few studies have examined oral tactile perceptions and food texture preferences in children [12,13].

Due to the paucity of methodological approaches to measure lingual tactile sensitivity in a younger population, the primary aim of the present study was to verify the cognitive and perceptive suitability of two different tools for tactile sensitivity evaluation (Von Frey filaments and gratings) in children of different ages (6–13 years). Our findings indicate that both tools are cognitively appropriate for children starting from the age of 6 years, as they were able to understand procedures and instructions and completed both tasks without major problems (see also Section 5 on methodological challenges of the study). From a perceptive point of view, we established that both tools can be used to measure lingual sensitivity in children between the ages of 6 to 13 years, as the R-index average values for the two filaments (F0.008 and F0.02) and the two higher gratings (G1.00 and G1.25) were greater than the respective cut-offs [47]. This indicates that the tools were sensitive enough to discriminate clearly between the two stimuli presented (signal vs. noise) [45], in children as well as adults. It should be underlined however that the gratings orientation task was far more difficult than the filament assessment for both children and adults.

The correlations analysis (Table 5) showed that sensitivity to the Von Frey filaments was not directly correlated with sensitivity to the gratings tool as well as suggested by the multivariate analysis (Figure 7), indicating a separation between the measurements taken by the two types of tools. The reason for this separation may be explained by the fact that Von Frey filaments and gratings, respectively, measure contact detection and spatial resolution sensitivity, which are recognized as distinctly different sensory functions subserved by different neural mechanisms [6,49,50]. Indeed, texture perception is rather complex and can be described by mechanical, geometrical and mouthfeel attributes [51], which are perceived by different mechanoreceptors [6]. Each property is likely coded by the mechanoreceptors through the combination of the signal patterns and integrated during higher processing in the brain to express the perceptions of specific basic textural modalities, such as smoothness, roughness or viscosity, all of importance to the eating experience [52]. In this context, Von Frey filaments can determine the minimum force that can be detected by the subject (touch detection threshold) and are the most commonly used devices to measure tactile sensitivity [53]. They reflect the response to a purely mechanical pressure, i.e., light touch detection, while the gratings evaluate the geometrical characteristics of texture perception [54]. Sensitivity to mechanical pressure in a discrete part of the tongue (e.g., as measured by the filaments) and sensitivity to spatial resolutions over a wider area (e.g., as measured by the gratings) reflect different phenomena, and hence, it is unsurprising that our results conclude that there is no correlation between the two.

Oral tactile sensitivity is reported to vary widely among individuals, and these population-wide differences have been suggested to depend on several factors, such as sex but, especially, age and dental status [27]. In the present study, children’s lingual tactile sensitivities were compared to that of adults. Anecdotal records suggested that children are more orally sensitive than adults. However, we found no age-related differences in the lingual tactile sensitivity performances among the different children’s age groups and adults. The comparable tactile acuity performances between the adults and children were previously reported by Lukasewycz and Mennella [13], who evaluated lingual acuity by the Essick’s letters recognition task [55] in a group of children aged 7–10 years and their mothers. The one exception in the present study was for the thinnest Von Frey filament (F0.008), for which the performances of children aged 8 to 9 years were significantly better than the younger children and adults. However, further studies are needed to draw consistent conclusions regarding differences in oral sensitivity between children and adults.

To the best of our knowledge, no studies have directly explored oral sensitivity and factors related to food consumption, such as texture preferences and food neophobia. In the present study, the MFA did not find a clear relationship between lingual tactile sensitivity and either CFTPQ or FNS. However, there was a significant negative correlation between higher tactile sensitivity measured using the finer filament and food neophobia in young children, suggesting a higher lingual tactile sensitivity could lead to a greater avoidance of new foods. This is somewhat in-line with the previous literature; for example, Smith and colleagues [56] showed that, in a group of children aged 3–10 years, tactile sensitivity was associated with a higher food texture aversion. This increased sensitivity was weakly related to higher neophobic attitudes, as previously suggested [18]. Indeed, Coulthard and Blisset [18] supported the hypothesis that tactile sensitivity is associated with food neophobia and pickiness in childhood, suggesting that texture perception in children could influence the rejection of certain foods. It has to be mentioned that the latter two mentioned studies evaluated children sensory processing abilities using questionnaires, which measure sensory processing in seven different domains, including tactile sensitivity (e.g., “Avoids going barefoot, especially in sand or grass”), and not actual measures of children’s sensory sensitivity. In this respect, the different approaches used to measure tactile sensitivity (either with questionnaires or tools) seem to provide comparable results. However, it should be underlined that lingual tactile sensitivity seems to play a controversial and unclear role in subjects’ decreased/increased preferences for, and frequency of, the consumption of foods of complex textures, supporting the assumption that, of the many factors contributing to food preferences and consumption, oral tactile sensitivity may not have the most immediate influence on food choice [28]. Indeed, texture is generally taken for granted, and consumers’ primary attention in making a choice are focused on the sight and the taste of a food, and the cognitive process is triggered by food texture properties only if the texture is definitely off or inappropriate, violating consumers’ expectations [1]. Moreover, the weak relationship found among the variables considered in the present study may be ascribed to the fact that lingual tactile sensitivity measured through Von Frey filaments and gratings might not reflect the complexity of texture perception, which implies dynamic processes such as chewing and swallowing thus resulting in low associations with eating behaviors. In this respect, foods usually deliver forces of higher orders of magnitude to the tongue than the von Frey filaments used in the study. Moreover, in contrast to the static pressure exerted by the gratings on the tongue, texture perception occurs when there is relative movement between the lingual mucosa and food substances. Thus, the use of tools that measure the ability to discriminate small differences in higher forces would seem more relevant to study the associations among oral tactile perception, texture preference and consumption in children, as well as adults.

The negative association between the CFTPQ index, food neophobia and consumption frequency of sweets (hard and soft), bakery (hard and soft) and potatoes (soft) is another interesting outcome of the present study, although only qualitative assumptions can be drawn. Since the CFTPQ index reflects the preferences for hard and lumpy foods, the findings that subjects (both adults and children, Figure 8a) with a greater level of food neophobia are those who prefer soft and homogeneous food textures seems realistic and in-line with the previous results [12,13]. Indeed, the disposition on the plot in Figure 8 of the soft and hard food versions seems to support this assumption. Food categories characterized by soft texture are positively associated with FNS, aside from bakery, sweets and potatoes, which are familiar and generally well-appreciated by children. Contrarily, a negative association between FNS and fruits (both hard and soft versions) was depicted, supporting the well-known neophobic children’s aversive attitudes for this food category.

## 5. Methodological Challenges of the Study

The methodological challenges encountered during the lingual tactile sensitivity assessment and suggestions for possible solutions are reported in Table 6. For both adults and children, we encountered several challenges in the application of both tools. If not taken into consideration, these challenges can influence the reliability of the test. In general, the reliability of the instruments is affected very much by the experimenter. Therefore, it is important to foresee a thorough training and calibration of the experimenters in order to exercise a standardized and constant force on the subject’s tongue. In addition, both instruments required considerable effort of concentration by the volunteers because of the duration of the test but, also, for the fact of having to keep the tongue stretched out for a relatively long time, which leads to involuntary movements of the lingual muscle and, also, to a dryness of the lingual surface. The occurrence of these limitations varies very much with individual variability but can be reduced by instructing the subject to keep the tongue relaxed between the teeth and possibly rest the chin on the hands keeping the arms leaning on a table.

It is interesting to note that, if properly educated and informed, a group of children can carry out the assessment successfully. The only problems we encountered with younger children (6-year-olds) referred to the reluctance to wear the blindfold for the entire duration of the evaluation. This obstacle was easily overcome by reassuring the child that she/he would be assisted all the times and that she/he would be allowed to remove the blindfold as long as they kept their eyes closed. Furthermore, young children were scared of the filaments, which, in some cases, were mistaken for needles. Additionally, in this case, before the start of the test, the experimenter showed the filaments’ flexibility and subsequently applied a demonstrative slight touch on the child’s hand.

The evaluation with the Von Frey filaments has two additional challenges, related to the extreme thinness of both filaments, so much that the breathing of the subject can cause the filament to move or bend, varying the intensity of the touch. In this case, it is important that the experimenter adjusts to the volunteer’s breathing rhythm or asks the volunteer to refrain from breathing for a moment. Furthermore, sitting aside of instead of facing the subject can improve the visibility of the filament.

## 6. Strengths and Limitations of the Study

A strength of the study is that it attempts to evaluate, for the first time, lingual tactile sensitivity in the pediatric population by using two different and practical approaches and compares it with a control group of adults. Moreover, the present research made important steps to shed some light on the association between tactile sensitivity and other background variables, which span from neophobia, preferences and frequency consumption of foods with different textures.

One limitation of the present study is that food texture preferences are not assessed using food stimuli but only via questionnaire. Moreover, since the evaluation of tactile sensitivity is still challenging, it would be of interest for future studies to investigate the reproducibility of the tactile sensitivity as measured by Von Frey filaments and grating orientation tasks. It has to be noted that a staircase approach or n-alternative forced choice method are also suitable to evaluate oral tactile thresholds. However, it is important to consider that these approaches could be affected by several biases when the pediatric population is involved in the evaluation. Indeed, our subjects, due to their young ages, have reduced cognitive/verbal skills, limited attention spans and may suffer of fatigue from routine procedures [57]. This makes the application of a staircase method, or any method requiring a higher number of trials to obtain a tactile threshold value, difficult. Such methods would be unfeasible with children who are required to stay blindfolded and to keep the tongue stretched out for a long time.

Moreover, the Von Frey filaments might not be a sufficiently sensitive tool to evaluate the oral tactile sensitivity, due to the fact that the lowest available force (0.08 mN) is higher than the sensitivity level of the tongue mucosa (0.015 mN) [58]. This may also have contributed to finding no age-related differences in tactile sensitivity. Different tongue sensitivity methods (e.g., Luneau Cochet-Bonnet aesthesiometers) [59] commonly used to measure corneal sensitivity could be employed to measure the touch detection sensitivity or thresholds, since these aesthesiometers have the benefit of providing an increased number of extremely lower-force stimuli than the Von Frey filaments. However, the present study was exploratory and the first to be conducted on a sample of children; therefore, Von Frey filaments were used based on the literature for an adult population [28,30,31,32]. Another limitation could be related to the custom-made grating orientation test used, which consists in flat-surface gratings, whereas the commercially available JVP probes are dome-shaped to improve contact with concave cutaneous surfaces (such as under the lower lip). However, the tongue is a relatively flat surface compared to other areas of the human body; thus, the use of the flat gratings is unlikely to be an overriding concern.

Another limitation is related to the limited number of participants. In fact, although the size of the population sample was satisfactory as a whole (*n* = 217: adults, *n* = 70 and children, *n* = 147), considerations about age-related differences were drawn on smaller numbers of subjects (approx. 30 for children). Future studies should confirm and extend our findings.

Finally, one important thing that should be highlighted is related to the R-index calculation, which is usually performed with a high number of presentations (*n* = 100–200) [44]. Such a large number of replications is not practical for the sensory analysis of food, but estimations of the R-index are still possible and have been shown to be practical with fewer replications (for example, 20 rather than 200). Smaller samples increase the variance of the R-index estimate, although, for many food science applications, this has not been a problem [45]. Yet, if the number of tastings/presentations is sufficiently large for volunteers to be able to detect the variation in the degree of difference between stimuli, as in the present case, the measure is useful [44]. Test-retest is recommended to substantiate the usefulness of estimates of sensitivity based on the limited numbers of presentations per individual (such as the six replicates of gratings used in this study). 

R-index values can be used to derive thresholds by extrapolating this value using the stimulus concentration/gratings width equivalent to an R-index of 75% or d’ value that corresponds to 75% of the correct responses (thresholds performance), e.g., [42]. However, the procedure has long- required several stimulus concentrations and replications, making it unsuitable for children due to the practical constraints previously cited. In the present study, the number of different grating sizes used for children (*n* = 3) was not appropriate for calculating a threshold.

It should be also considered that one of the aims of the present study was to test the suitability of the two methods (filaments vs. gratings) to assess subjects’ tactile sensitivity, which increased the complexity of the task for children (and adults, too). If only one approach is used (e.g. gratings), then a higher number of trials could be performed, perhaps splitting the test into several sessions. However, this is not always feasible when testing children in ecological environments such as a school.

## 7. Conclusions

In conclusion, the findings of this study provided information about suitable methodological approaches to measure oral tactile sensitivity in children. Both Von Frey filaments and the gratings orientation task can be used to measure the lingual sensitivity in 6- to 13-year-old children, as well as in adults. Gratings enabled a better separation of subjects according to their discrimination ability, although the task was more difficult than the filament assessment. As filaments and gratings appear to provide a separate measure of lingual sensitivity, it might be wise to utilize both instruments in future research studies that aim to relate the sensitivity of the lingual tactile response to food perception and food choice. The availability of these child-friendly methods for measuring oral tactile sensitivity opens new opportunities in the study of population differences in texture perceptions across the lifespan.

In the present study, we did not find any age-related differences in the lingual tactile sensitivity performance among children with different ages and adults. Considering that healthy people greatly vary in tactile sensory function on the anterior tongue, differences in food texture perception, consumer preference and the consumption of foods varying in texture are expected. Understanding these relationships is of interest to food scientists, as it is relevant to the development of foods that are healthy, preferred and consumed by different population targets. Further studies are needed to investigate the relationship between oral sensitivity and food texture.

This study reveals—even if only in a qualitative manner—a link between lingual tactile sensitivity, food texture preference and food neophobia. Thus, we suggest that early life experiences with texture varieties are necessary to encourage children’s acceptance and consumption of harder foods or foods containing particles.

The present results could be of interest to food science research and to the food industry, which aims to develop food products where the improved texture is required.

## Figures and Tables

**Table 1:** Characteristics of the participants and tasks performed in the study.

Participants	N	Sex (% Females)	Age (mean ± s.d.)	Age Range (Years)	Task Performed
Children	147	49.7	9.2 ± 2.2	6–13	Lingual tactile assessment (Von Frey filaments and Gratings orientation task) + questionnaires (CFTPQ ^a^ and ChFNS ^b^)
Parents	65	76.9	44.3 ± 5.7	32–58	Questionnaire (FFQ ^c^) relating to their child’s intake
Adults	70	52.9	22.0 ± 3.4	19–33	Lingual tactile assessment (Von Frey filaments and Gratings orientation task) + questionnaires (CFTPQ, FNS ^d^ and FFQ)

^a^ CFTPQ: Child Food Texture Preference Questionnaire [12], ^b^ ChFNS: Child Food Neophobia Scale [34], ^c^ FFQ: Food Consumption Frequency Questionnaire [12] and ^d^ FNS: Food Neophobia Scale [35].

**Table 2:** Categorization of food items according to texture.

Food Category	Texture Version	Food Item
Bakery products	Hard	Toasted bread, crackers, cornflakes
	Soft	White bread, cookie with melted filling, soaked cornflakes
Dried fruits and nuts	Hard	Dried fruits and nuts
Fruits	Hard	Apple, peach, grape
	Soft	Banana, kiwi, melon, apple puree
Vegetables	Hard	Raw carrot, raw fennel, raw bell pepper, raw celery, cherry tomato
	Soft	Cooked carrot, cooked zucchini, cooked bell pepper, cooked fennel, tomato sauce, peas, bean, lentils
Potatoes	Hard	Boiled potato
	Soft	Potato puree
Dairy products	Hard	Firm cheese
	Soft	Spreadable cheese
Sweets	Hard	Hard candies, lollipop, ice lolly, chocolate bar, crunchy ice cream
	Soft	Marshmallow, soft candies, soft ice cream, ice slush, chocolate pudding

**Table 3:** Dimensions of grooves used in the gratings orientation test.

Groove Width (mm)	Bar Width (mm)	Groove Depth (mm)
0.20	0.20	0.30
0.25	0.25	0.40
0.50	0.50	0.75
0.75	0.75	1.15
1.00	1.00	1.50
1.25	1.25	1.95

**Table 4:** Example of the response matrix used to collect data from Von Frey filaments (F) and the gratings orientation (G) test (S = signal and N = noise).

Stimulus (Filaments/Gratings)	Response Options			
	S, Sure	S, Unsure	N, Unsure	N, Sure
Signal (F: true touch; G: horizontal)	a	b	c	d
Noise (F: false touch; G: vertical)	e	f	g	h

**Table 5:** Spearman’s correlation coefficients between tools in the total samples of the subjects (*n* = 217). * *p* < 0.05, ** *p* < 0.01 and ***p < 0.001.

Tool	F0.008	F0.02	G0.75	G1.00	G1.25
**F0.008**	1				
**F0.02**	0.44 ***	1			
**G0.75**	0.22 **	0.17 *	1		
**G1.00**	0.15 *	0.09	0.25 ***	1	
**G1.25**	0.13	0.09	0.35 ***	0.29 ***	1

**Table 6:** Summary of the methodological challenges encountered in the evaluation of lingual tactile sensitivity in children and adults and possible solutions.

Tool	Methodological Challenges	Who	Recommendations
Von Frey filaments	The extreme filament thinness is influenced by subject’s breath varying the intensity of touch perception	Both adults and children	Pay close attention to the subject’s breathing rhythm or ask to refrain breathing for a moment
Von Frey filaments	Filaments are almost invisible	Both adults and children	Sit next to the subject and to one side
Von Frey filaments	Filament mistaken for a needle	Children	Make a demonstrative touch on the child’s hand
Both	The touch is subject to the variability of the experimenter	Both adults and children	Experimenter training and calibration
Both	Subjects involuntarily move their tongue during the evaluation	Both adults and children	Instruct subjects to keep the tongue relaxed between the teeth and rest the chin on the hands, keeping the arms leaning on a table
Both	Dry mouth	Both adults and children	Take breaks and drink water
Both	Reluctance in holding the blindfold during the entire evaluation	Children	Remove the blindfold, ensuring that the eyes remain closed
Both	Loss of attention	Children aged 6–7 years	Create a game-like situation. Split the evaluation into two rounds

## References

[1] Szczesniak A.S. (2002). Texture is a sensory property. Food Qual. Prefer..

[2] Darian-Smith I. (2011). The sense of touch: Performance and peripheral neural processes. Compr. Physiol..

[3] Jones L.A. (1994). Peripheral mechanisms of touch and proprioception. Can. J. Physiol. Pharmacol..

[4] Johnson K.O., Yoshioka T., Vega–Bermudez F. (2000). Tactile functions of mechanoreceptive afferents innervating the hand. J. Clin. Neurophysiol..

[5] Matthews P.B. (1988). Proprioceptors and their contribution to somatosensory mapping; complex messages require complex processing. Can. J. Physiol. Pharmacol..

[6] Abraira V.E., Ginty D.D. (2013). The sensory neurons of touch. Neuron.

[7] Trulsson M., Essick G.K. (2010). Sensations evoked by microstimulation of single mechanoreceptive afferents innervating the human face and mouth. J. Neurophysiol..

[8] Szczesniak A.S. (1972). Consumer awareness of and attitudes to food texture II. Children and teenagers. J. Texture Stud..

[9] Scott C.L., Downey R.G. (2007). Types of food aversions: Animal, vegetable, and texture. J. Psychol..

[10] Werthmann J., Jansen A., Havermans R., Nederkoorn C., Kremers S., Roefs A. (2015). Bits and pieces. Food texture influences food acceptance in young children. Appetite.

[11] Jeltema M., Beckley J., Vahalik J. (2016). Food texture assessment and preference based on Mouth Behavior. Food Qual. Prefer..

[12] Laureati M., Sandvik P., Almli V.L., Sandell M., Zeinstra G.G., Methven L., Wallner M., Jilani H., Alfaro B., Proserpio C. (2020). Individual differences in texture preferences among European children: Development and validation of the Child Food Texture Preference Questionnaire (CFTPQ). Food Qual. Prefer..

[13] Lukasewycz L.D., Mennella J.A. (2012). Lingual tactile acuity and food texture preferences among children and their mothers. Food Qual. Prefer..

[14] Rose G., Laing D.G., Hutchinson O.I. (2004). Sensory profiling by children aged 6–7 and 10–11 years. Part 1: A descriptor approach. Food Qual. Prefer..

[15] Zeinstra G.G., Koelen M.A., Kok F.J., de Graaf C. (2010). The influence of preparation method on children’s liking for vegetables. Food Qual. Prefer..

[16] Laureati M., Cattaneo C., Lavelli V., Bergamaschi V., Riso P., Pagliarini E. (2017). Application of the check-all-that-apply method (CATA) to get insights on children’s drivers of liking of fiber-enriched apple purees. J. Sens. Stud..

[17] Blossfeld I., Collins A., Kiely M., Delahunty C. (2007). Texture preferences of 12-month-old infants and the role of early experiences. Food Qual. Prefer..

[18] Coulthard H., Blissett J. (2009). Fruit and vegetable consumption in children and their mothers. Moderating effects of child sensory sensitivity. Appetite.

[19] Lundy B., Field T., Carraway K., Hart S., Malphurs J., Rosenstein M., Pelaez-Nogueras M., Coletta F., Ott D., Hernandez-Reif M. (1998). Food texture preferences in infants versus toddlers. Early Child Dev. Care.

[20] Lafraire J., Rioux C., Giboreau A., Picard D. (2016). Food rejections in children: Cognitive and social/environmental factors involved in food neophobia and picky/fussy eating behavior. Appetite.

[21] Coulthard H., Thakker D. (2015). Enjoyment of tactile play is associated with lower food neophobia in preschool children. J. Acad. Nutr. Diet.

[22] Wardle J., Herrera M.L., Cooke L., Gibson E.L. (2003). Modifying children’s food preferences. The effects of exposure and reward on acceptance of an unfamiliar vegetable. Eur. J. Clin. Nutr..

[23] Anez E., Remington A., Wardle J., Cooke L. (2013). The impact of instrumental feeding on children’s responses to taste exposure. J. Hum. Nutr. Diet..

[24] Fildes A., van Jaarsveld C.H., Wardle J., Cooke L. (2013). Parent-administered exposure to increase children’s vegetable acceptance. A randomized controlled trial. J. Acad. Nutr. Diet..

[25] Laureati M., Cattaneo C., Bergamaschi V., Proserpio C., Pagliarini E. (2016). School children preferences for fish formulations: The impact of child and parental food neophobia. J. Sens. Stud..

[26] Essick G.K., Chen C.C., Kelly D.G. (1999). A letter-recognition task to assess lingual tactile acuity. J. Oral. Maxillofac. Surg..

[27] Bangcuyo R.G., Simons C.T. (2017). Lingual tactile sensitivity: Effect of age group, sex, and fungiform papillae density. Exp. Brain Res..

[28] Cattaneo C., Liu J., Bech C.A., Pagliarini E., Bredie W.L.P. (2020). Cross-cultural differences in lingual tactile acuity, taste sensitivity phenotypical markers and preferred oral processing behaviors. Food Qual. Prefer..

[29] Bell-Krotoski J.A. (2011). Sensibility Testing: History, Instrumentation and Clinical Procedures. Rehabilitation of the Hand and Upper Extremity.

[30] Breen S.P., Etter N.M., Ziegler G.R., Hayes J.E. (2019). Oral somatosensatory acuity is related to particle size perception in chocolate. Sci. Rep..

[31] Santagiuliana M., Marigómez I.S., Broers L., Hayes J.E., Piqueras-Fiszman B., Scholten E., Stieger M. (2019). Exploring variability in detection thresholds of microparticles through participant characteristics. Food Funct..

[32] Yackinous C., Guinard J.X. (2001). Relation between PROP taster status and fat perception, touch, and olfaction. Physiol. Behav..

[33] Van Boven R.W., Johnson K.O. (1994). The limit of tactile spatial resolution in humans Grating orientation discrimination at the lip, tongue, and finger. Neurology.

[34] Laureati M., Bergamaschi V., Pagliarini E. (2015). Assessing childhood food neophobia: Validation of a scale in Italian primary school children. Food Qual. Prefer..

[35] Pliner P., Hobden K. (1992). Development of a scale to measure the trait of food neophobia in humans. Appetite.

[36] Laureati M., Pagliarini E., Ares G., Varela P. (2018). New developments in sensory and consumer research with children. Alternative Approaches and Special Applications.

[37] Kimmel S., Sigman-Grant M.J., Guinard J.-X. (1994). Sensory testing with young children. Food Technol..

[38] Laureati M., Spinelli S., Monteleone E., Dinnella C., Prescott J., Cattaneo C., Proserpio C., De Toffoli A., Gasperi F., Endrizzi I. (2018). Associations between food neophobia and responsiveness to “warning” chemosensory sensations in food products in a large population sample. Food Qual. Prefer..

[39] Peterson W.W., Birdsall T.G., Fox W.C. (1954). The theory of signal detection theory. IEEE Trans. Inf. Theory..

[40] Tanner W.P., Swets J.A. (1954). A decision-making theory of visual detection. Psychol. Rev..

[41] Swets J.A., Tanner W.P., Birdsall T.G. (1961). Decision processes in perception. Psychol. Rev..

[42] Kappes S.M., Schmidt S.J., Lee S.Y. (2006). Mouthfeel detection threshold and instrumental viscosity of sucrose and high fructose corn syrup solutions. J. Food Sci..

[43] Robinson K.M., Klein B.P., Lee S.Y. (2004). Utilizing the R-index measure for threshold testing in model caffeine solutions. Food Qual. Prefer..

[44] O’Mahony M. (1992). Understanding discrimination tests: A user-friendly treatment of response bias, rating and ranking R-index tests and their relationship to signal detection. J. Sens. Stud..

[45] Lee H.S., Van Hout D. (2009). Quantification of sensory and food quality: The R-index analysis. J. Food Sci..

[46] Berquin A.D., Lijesevic V., Blond S., Plaghki L. (2010). An adaptive procedure for routine measurement of light-touch sensitivity threshold. Muscle Nerve.

[47] Bi J., O’Mahony M. (2020). R-index critical value. J. Sens. Stud..

[48] Spence C., Hobkinson C., Gallace A., Fiszman B.P. (2013). A touch of gastronomy. Flavour.

[49] Johnson K.O., Phillips J.R. (1981). Tactile spatial resolution. I. Two-point discrimination, gap detection, grating resolution, and letter recognition. J. Neurophysiol..

[50] Phillips J.R., Johnson K.O. (1981). Tactile spatial resolution. II. Neural representation of bars, edges, and gratings in monkey primary afferents. J. Neurophysiol..

[51] Szczesniak A.S. (1963). Classification of Textural Characteristics. J. Food Sci..

[52] Foegeding E.A., Vinyard C.J., Essick G., Guest S., Campbell C. (2015). Transforming structural breakdown into sensory perception of texture. J. Texture Stud..

[53] Wiggermann N.E., Werner R.A., Keyserling W.M. (2012). The effect of prolonged standing on touch sensitivity threshold of the foot: A pilot study. PM&R.

[54] Koç H., Vinyard C.J., Essick G.K., Foegeding E.A. (2013). Food oral processing: Conversion of food structure to textural perception. Annu. Rev. Food Sci. Technol..

[55] Essick G.K., Chopra A., Guest S., McGlone F. (2003). Lingual tactile acuity, taste perception, and the density and diameter of fungiform papillae in female subjects. Physiol. Behav..

[56] Smith A.M., Roux S., Naidoo N.R., Venter D.J. (2005). Food choices of tactile defensive children. Nutrition.

[57] Guinard J.X. (2000). Sensory and consumer testing with children. Trends Food Sci. Tech..

[58] Trulsson M., Essick G.K. (1997). Low-threshold mechanoreceptive afferents in the human lingual nerve. J Neurophysiol..

[59] Miles B.L., Van Simaeys K., Whitecotton M., Simons C.T. (2018). Comparative tactile sensitivity of the fingertip and apical tongue using complex and pure tactile tasks. Physiol. Behav..

